# Immunization Against Active Ghrelin Using Virus-Like Particles for Obesity Treatment

**DOI:** 10.2174/13816128113199990506

**Published:** 2013-11

**Authors:** Sara Andrade, Filipa Pinho, Andreia M Ribeiro, Marcos Carreira, Felipe F Casanueva, Polly Roy, Mariana P Monteiro

**Affiliations:** 1 Department of Anatomy and UMIB (Unit for Multidisciplinary Biomedical Research) of ICBAS, University of Porto, Portugal;; 2 CIBER de FisiopatologiaObesidad y Nutricion (CB06/03), InstitutoSalud Carlos III, Santiago de Compostela, Spain;; 3 Department of Medicine, USC University Hospital Complex, University of Santiago de Compostela, Santiago de Compostela, Spain; 4 Department of Pathogen Molecular Biology, London School of Hygiene and Tropical Medicine, London, UK

**Keywords:** obesity, ghrelin, vaccine, virus-like particles, treatment.

## Abstract

Ghrelin is a gut hormone that stimulates food intake. In physiological conditions, ghrelin plasma levels rise with fasting and decrease after meals. Obese individuals have low fasting ghrelin levels that rise after food restriction, which is pointed out as a reason for the difficulty in maintaining weight loss. Some bariatric surgery procedures prevent rise in ghrelin levels with weight loss and this has been hypothesised to contribute to the long-term success of the treatment.

The main goal of this study was to develop a safe and effective anti-ghrelin vaccine for obesity, through the chemical conjugation of ghrelin with a virus like particle, namely NS1 protein tubules from the Bluetongue Virus (BTV) using a hetero-bifunctional cross linker.

Male adult C57BL/6 mice, with a normal weight and with diet-induced obesity (DIO), were randomized into six weight matched groups (n=6/group) and each group of mice received three intra-peritoneal injections with two weeks intervals, containing either 75 µg of ghrelin-NS1 immunoconjugate, 75 µg of NS1 or PBS. Our data show that immunized animals present increasing titres of anti-ghrelin antibodies, while their cumulative food intake significantly decreased and energy expenditure was significantly enhanced, although there were no significative changes in body weight.Vaccinated DIO mice also displayed significant decrease of NPY gene expression in the basal hypothalamus reflecting a decrease in central orexigenic signals.

This study suggests that this anti-ghrelin vaccine has a positive impact on energy homeostasis and may be an additional therapeutical tool to be used with diet and exercise for obesity treatment.

## 1 INTRODUCTION 

Obesity is increasing worldwide [1, 2]. Obesity is associated with enhanced prevalence of several co-morbidities such as type 2 diabetes mellitus, metabolic syndrome and cardiovascular diseases [3, 4] and weight loss improves or resolves several co-morbid conditions associated with the disorder [5]. Clinicians have few tools to fight obesity. Diet and exercise are still the cornerstone for obesity treatment and current anti-obesity drugs achieve only relative short-term weight loss [6] and are often followed by weight regain [7]. For morbid obesity, bariatric surgery is the only therapy that provides sustainable weight reduction [8]. Recent studies, suggest that weight loss attained after bariatric surgery is also due to endocrine effects of the surgery, which are able to interfere with appetite pathways by suppressing the rise in ghrelin levels that is usually observed after caloric deprivation [9, 10].

As a proof of this concept, it has been demonstrated that inoculation of monoclonal anti-ghrelin antibodies in mice inhibited acute ghrelin-mediated orexigenic effects but it was unable to change long-term food intake [11]. More recently, another study suggested that the use of a mixture of monoclonal antibodies targeting different haptens, but not the antibodies individually, promotes not only an increase in energy expenditure but also a reduced deprivation-induced food intake [12]. Ghrelin receptor antagonists, GSH-R1demonstrated improved glucose tolerance, suppressed appetite and promoted weight loss [13], thus confirming the potential of ghrelin blocking as a potential treatment target for obesity. The suppression of endogenous ghrelin bioactivity with anti-ghrelin vaccines using keyhole limpet hemocyanin (KLH) as carrier protein [14] or bovine serum albumin (BSA) [15], were also tested in mice and pigs, respectively. These vaccines were able to induce the development of antibodies against the active form of ghrelin [14] and also to decrease body weight gain and fat mass [15]. However, these vaccines required the use of adjuvants, such as alum and Freund incomplete adjuvant, which may be associated of inflammatory responses or have limited use in humans.

Virus-like particles (VLPs) have been used as immunogenic molecules in several recombinant vaccines in the last few years in order to induce the production of specific antibodies against endogenous molecules with a preponderant role in chronic diseases [16], such as the anti-angiotensin vaccine developed for arterial hypertension treatment [17]. 

The main goal of this study was to develop an effective anti-ghrelin vaccine using a chemical conjugate of active ghrelin with protein tubules of NS1 of the Bluetongue Virus (BTV) [18]. Although this protein is not part of the viral capsid, NS1 tubules possess the same immunogenic characteristics as classical VLPs [19]. 

## 2 MATERIALS AND METHODS

### 2.1 Imunoconjugate Production

Tubules of NS1 protein of BTV were chemically conjugated to active ghrelin using the heterobifunctional cross linker EDC (Pierce, Illinois, USA), by incubating NS1 with an excess of EDC and N-hydroxysuccinimide (NHS), which activates the carboxyl groups present in the surface of the NS1 tubules. Ghrelin (H4862, Bachem, Germany) was added in excess and left to react at room temperature for 2 hours and the reaction was stopped by adding hydroxylamine. The cross linker in excess was removed by gel filtration chromatography using Zeba Columns (Pierce, Illinois, USA). The newly formed immunoconjugate was recovered by dialysis in phosphate buffer solution (PBS) and the efficacy of the conjugation was assessed by SDS-PAGE.

### 2.2 Animals

Thirty-six male adult C57BL6/J mice (Charles River, Barcelona, Spain), normal weight and with diet induced obesity mice (DIO) (n=18/group), were randomized into three weight-matched groups (n=6/group). Mice were maintained in standard animal house conditions with constant temperature (21±1ºC), humidity and 12h controlled photoperiod (7h-19h). Normal weight mice were housed in groups (n=6 mice/cage) and DIO mice were housed individually. Normal weight mice had unrestricted access to tap water and regular rat chow (SAFE – Scientific Animal Food & Engineering) and DIO mice to a hypercaloric diet with 60% of fat (Charles River, Barcelona, Spain), after weaning and until a week before the first immunization study when food was switched to regular rat chow (SAFE – Scientific Animal Food & Engineering). All procedures were approved by the local Ethics Board for Animal Research and followed the European Union laws on animal protection (86/609/EC).

### 2.3 Immunization Protocols

Mice received three intra-peritoneal (i.p.) injections with two week intervals, containing 500μl of 75μg of immunoconjugate, 75μg of NS1 protein alone or PBS. The additional control group inoculated with NSI only was included in order to distinguish the effects of the immune complex from NS1 ALONE. 

A dose chosen after performing a dose-response study (data not shown) in which the 75 μg dose of the immuneconjugate has demonstrated to be adequate in inducing the development of anti-ghrelin antibodies and reduce food intake. 

Food intake was evaluated daily by weighing the remaining food in the hopper and 1, 2, 4, 8, 16 and 24h after each immunization using a scale recording to the nearest 0.1 g (KB 5000-1, Kern, Germany). Body weight was assessed twice a week using a scale recording to the nearest 1 g (Monobloc, Mettler-Toledo, Switzerland). Mice were sacrificed two weeks after the last immunization.

### 2.4 Evaluation of the Orexigenic Response to Exogenous Ghrelin

To evaluate the response to exogenous ghrelin, DIO mice received 10 μg of ghrelin (H4862, Bachem, Germany) i.p. or PBS on different occasions, while food intake was accessed 30, 60, 120 and 180 minutes after the injections. These studies were performed before the start of the inoculation protocol while on hypercaloric diet and repeated a week after the introduction of standard diet. 

### 2.5 Evaluation of energy expenditure

Energy expenditure was accessed by indirect calorimetry. Mice were individually placed in a small grid cage to limit locomotor activity, which was placed into a sealed chamber containing a sodium hydroxide recipient to adsorb carbon dioxide. The lid of the chamber was sealed and pierced by a volumetric pipette to measure the volume of oxygen consumed. The time elapsed until 1ml was consumed was registered and repeated until 5 concordant values were obtained. The energy expenditure was then calculated considering that 4.82 kcal is the average energy released per litre of O_2_ consumed.

### 2.6 Anti-ghrelin Antibodies Titre Assessment

Anti-ghrelin antibodies titre was determined two weeks after each immunization in the serum of blood collected by submandibular vein puncture, by *in house* ELISA. Wells were coated with a 3μg/ml solution of ghrelin (H4862, Bachem, Germany) and the blocking of was done with TST+2%BSA solution. Serum samples were serially diluted in TST (10mM Tris (pH8.0), 150 mMNaCl, 0.05% Tween 20) starting with a 1:30 dilution until a 1:3 dilution. Samples were allowed to react in the wells for 2 hours at room temperature and IgG secondary antibody conjugated to Alcaline Phosphatase (1030-04, Southern Biotech, Birmingham, USA) in a 1:500 dilution was then added for 1 hour. The wells were washed and the substrate *p-*p-Nitrophenyl phosphate in AP buffer (50mM Na_2_CO_3_, 1mM MgCl_2_, pH 9.8) was added and the reaction was stopped with 0.1 M EDTA (pH=8.0) solution. Absorbances were read immediately after the addition of the stopping solution at a wavelength of 405 nm. Antibody titres were determined from the curves Abs= *f* (dilution).

### 2.7 Hormone Measurements

At the end of the experiments, 12h fasted mice were anesthetized with CO_2_ and blood was collected by cardiac puncture into chilled lithium heparin tubes containing a protease inhibitor (0.02 ml Trasylol, Bayer, Portugal). Tubes were kept on ice and immediately centrifuged at 4ºC. Plasma was stored at -20ºC until the assays where performed. Plasma levels of active ghrelin (EZRGRA-90K, Linco Research, St. Charles, Mo, USA, range 25 - 2000 pg/mL), leptin (EZML-82K, Linco Research, St. Charles, Mo, USA, range 0.2 - 30 ng/ml), insulin (EZRMI-13K, Linco Research, St. Charles, Mo, USA, range 0.2 - 10 ng/ml), growth hormone (EZRMGH-45K, Linco Research, St. Charles, Mo, USA, range 0.07–50 ng/mL), IGF-1 (E25, Mediagnost, Reutlingen, Germany, range 0.5 - 18 ng/ml) and TNF-α (Quantikine, R&D Systems, Abingdon, United Kingdom, range 15.6 - 1000 pg/ml) were determined by ELISA using specific commercial kits according to the manufacturer instructions. Blood glucose levels were analysed in the whole blood by the glucose oxidase method using a glucometer (One Touch Ultra, Lifescan, Johnson and Johnson, Milipitas, CA). Epididymal fat pads were also collected and weighed.

### 2.8 Test for the Presence of Circulating and Deposits of Immune Complexes

Microplate wells were coated with a 3 μg/ ml solution of anti-mouse ghrelin rabbit IgGs (Bachem, California, USA) and were kept overnight at 4º C. Blocking was done by addition of a TST+2%BSA solution. Serum samples were allowed to react in the wells for 2 hours at room temperature and then washed in TST 10mM Tris (pH8.0), 150 mMNaCl, 0.05% Tween 20) buffer. The IgG secondary antibody conjugated to Alcaline Phosphatase (1030-04, Southern Biotech, Birmingham, USA) was added at a 1:1000 dilution and allowed to react for 1 hour. The substrate *p-*p-Nitrophenyl phosphate in AP buffer (50mM Na_2_CO_3_, 1mM MgCl_2_, pH 9.8) was then added and the reaction was stopped with a 0.1 M EDTA solution.

To detect the presence of immune complexes deposits in the glomerular membrane, kidneys were collected, fixated in 10% buffered formaldehyde, processed for inclusion in paraffin blocks and an immunohistochemisty for immunoglobulins using the avidin-biotin-peroxidase (ABC) modified method was performed. Briefly, the endogenous peroxidases were blocked with a 0.3 solution of hydrogen peroxide in methanol, followed by incubation in a moist chamber with 1% BSA (Sigma-Aldrich, St. Louis, USA). Excess BSA was removed and microscope slides were incubated with rabbit anti-mouse antiserum (E0354, DakoCytomation, Denmark) at a 1:200 dilution followed by the avidin-biotin-peroxidase complex (DakoCytomation, Denmark) and the reaction was revealed with diaminobenzidine. Finally, the slides were coloured with hematoxylin and mounted for observation on optic microscope. Spleens of the same animals were used as positive controls due to their constant baseline production of antibodies.

### 2.9 RNA Extraction and Real Time PCR

Two weeks after the third immunization, mice were sacrificed and the stomach fundus and the hypothalamus were recovered and immediately frozen by immersion in liquid nitrogen to evaluate ghrelin, Neuropeptide Y (NPY) and Proopiomelanocortin (POMC) expression. 

Total RNA was isolated from using RNeasy Mini Kit (Qiagen, Germany) according to the manufacturer’s instructions and 500ng of RNA was retrotranscribed into cDNA using High Capacity cDNA Reverse Transcription Kit (Applied Biosystems, Foster City, CA).

The RNA expression of ghrelin, NPY and POMC in hypothalamus and/or stomach were studied by using TaqMan real-time PCR in Step One Plus system (Applied Biosystems, Foster City, CA) using specific primers and probes obtained from inventoried TaqMan Gene Expression Assays (Applied Biosystems, Foster City, CA). All reactions were carried out using the following cycling parameters: 50 C for 2 min, 95 C for 10 min followed by 40 cycles of 95 C for 15 sec, 60 C for 1 min. For the analysis of the data, the RNA level of the gene of interest was normalizing using 18S GADPH for stomach and β-actin for hypothalamus values according with the 2^-ΔΔCt ^method.

### 2.10 Statistical Analysis

Statistical analysis was made using SPSS statistical package for Windows version 18.0. Comparison of the means between groups was made using One-Way ANOVA with post hoc Bonferroni correction or Kruskall-Wallis tests, as appropriate. Pearson test was used for the correlation study. Results are shown as mean ± standard error (Mean±SEM), unless otherwise specified. A p value of <0.05 was considered statistically significant. 

## 3 RESULTS

### 3.1 Immunoconjugate Production 

**Fig. (1):**
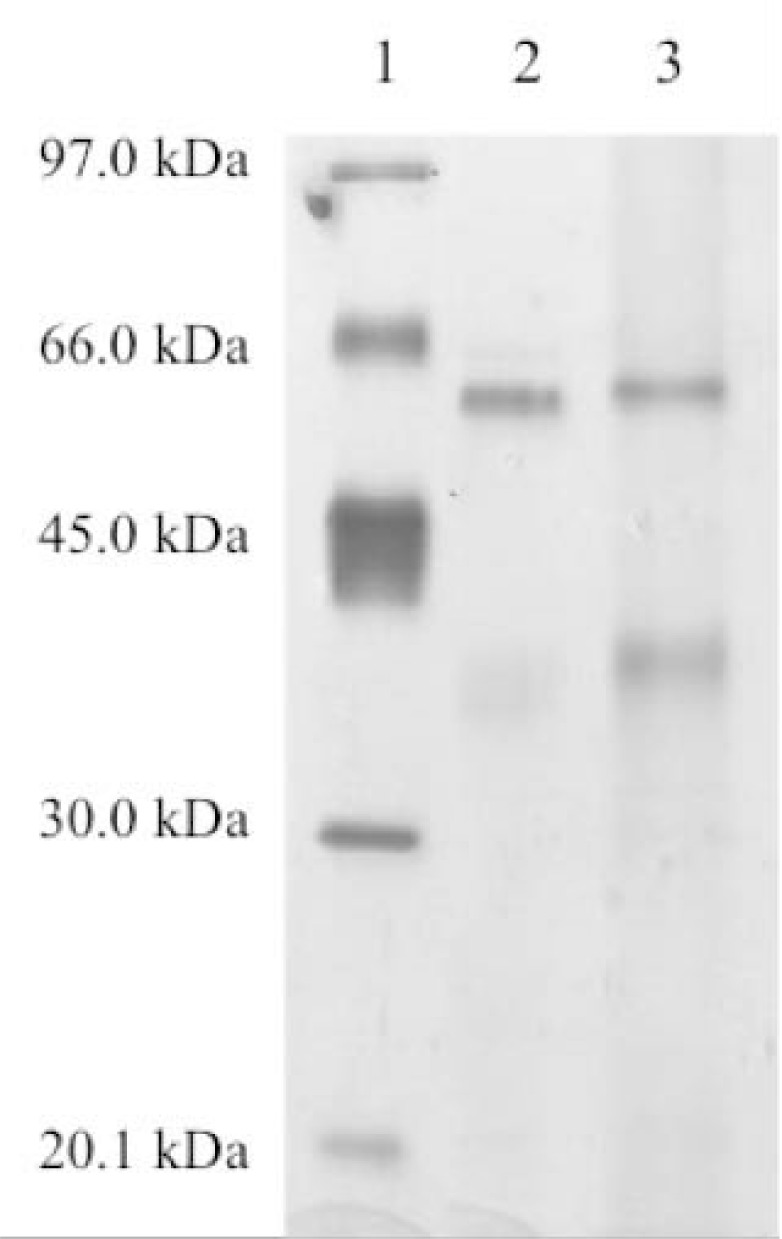
Silver stained SDS-PAGE showing Low Molecular Weight Markers on lane 1, NS1 protein alone on lane 2 and immunoconjugate on lane 3.

The immunoconjugate of active ghrelin and NS1 protein of BTV was produced and revealed in the SDS-PAGE by the slightly higher molecular weight of the immunoconjugate as compared to NS1 protein alone due to the low molecular weight of ghrelin (3.314 kDa) (Fig. **1**).

### 3.2 *In vivo* Studies 

#### 3.2.1 Food Intake and Body Weight 

**Fig. (2):**
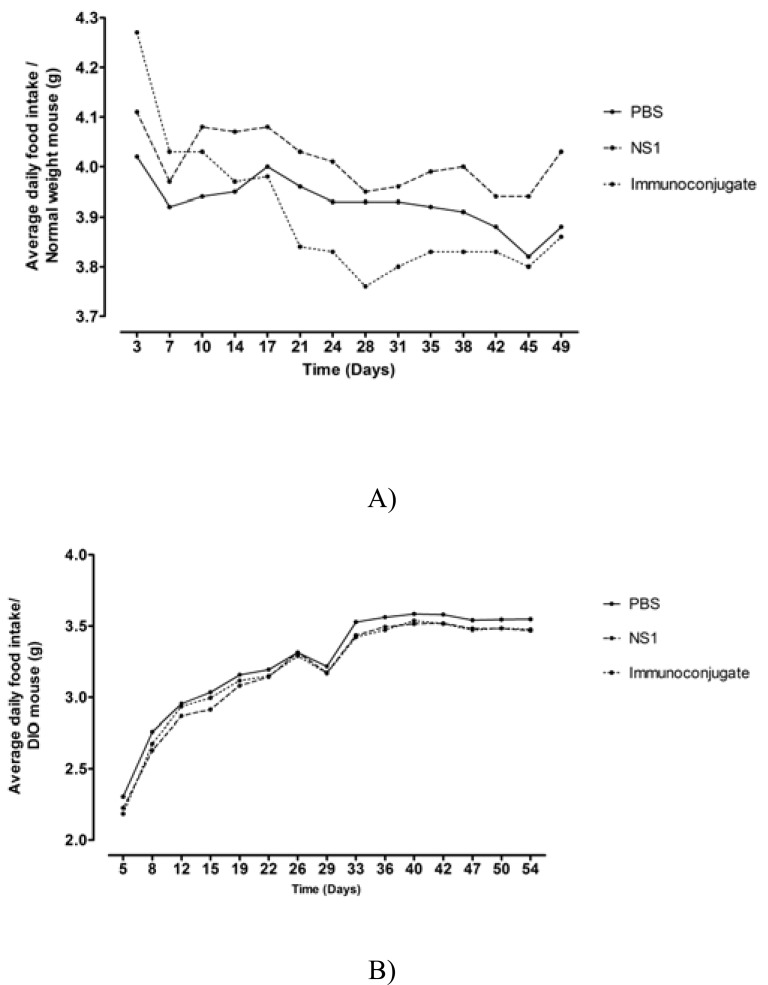
Mean daily food intake of normal weight (**A**) and DIO mice (**B**). Normal weight mice vaccinated with the immunoconjugate displayed a significant decrease in daily food intake (**A**); while DIO mice, displayed an initial increase in daily food intake after changing of hypercaloric to standard diet, which was followed by stabilization, there was no significative difference between vaccinated and control mice (**B**).

**Fig. (3):**
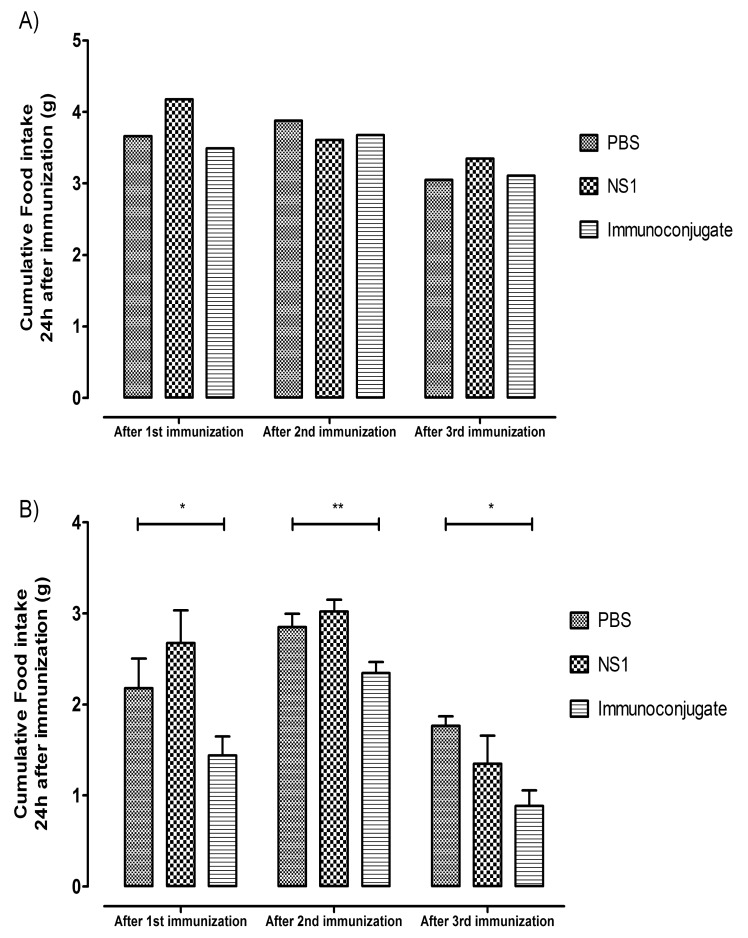
Graph displaying cumulative food intake in the first 24h after each immunization in normal weight (**A**) and DIO mice (**B**). Vaccinated normal weight mice, after the first two inoculations, depicted an acute decrease in food intake when compared to the PBS controls, corresponding to 95.3% and 94.8% of the PBS control, respectively (**A**). Vaccinated DIO mice also displayed a significant decrease of food intake in the first 24 hours after each inoculation of the immunoconjugate, corresponding to 66.16% (p=0.036), 82.22% (p=0.008) and 50.09 % (p=0.039) of the food intake of the PBS control group, respectively (**B**).

Normal weight mice treated with the immunoconjugate displayed a significant decrease in daily food intake (0.44 g NS1-Ghr *vs* 0.14 g PBS *vs* 0.16 g NS1, p<0.001, since the animals were group housed per cage there are no errors attached to the values) (Fig. **2A**). In addition, after the first two inoculations, there was also an acute decrease, although without reaching statistical significance, in food intake in the group of mice that received the immune conjugate when compared to the PBS control, corresponding to 95.3% and 94.8% of the PBS control, respectively (Fig. **3A**). There were no significant differences in body weight gain between the different groups of mice during the study span (3.83 g ± 0.40 g NS1-Ghr *vs* 5.00 g ± 0.26 g PBS *vs* 5.33 g ± 0.49 g NS1, p=NS).

In DIO mice, after changing from the hypercaloric to the standard diet, there was an increase in daily food intake followed by rapid stabilization (Fig. **2B**). DIO mice inoculated with the immunoconjugate did not display a significant decrease in cumulative food intake when compared to controls (147.64±2.46 g NS1-Ghr, 147.80±5.89 g NS1, 150.37±3.65 g PBS, p=NS), although there was a significant decrease of food intake in the 24 hours immediately after each inoculation of the immunoconjugate, corresponding to 66.16% (p=0.036), 82.22% (p=0.008) and 50.09 % (p=0.039) (Fig. **3B**) of the food intake of the PBS group, after the three inoculations, respectively. DIO mice body weight decreased in response to the change from the hypercaloric to the standard diet (13.84% compared to baseline), although after the inoculations, there were no significant differences in body weight among the different experimental groups (32.17 ± 0.872 g NS1-Ghr *vs* 31.33 ± 1.282 g NS1 vs 31.83 ± 0.833 g PBS, p=NS). 

#### 3.2.2 Evaluation of the Orexigenic Response to Exogenous Ghrelin

DIO mice, while on a hypercaloric diet, did not present any significant increase in food intake after the administration of exogenous ghrelin. However, one week after the introduction of the standard diet, DIO mice showed a significant increase in food intake in the first hour after the administration of exogenous ghrelin when compared to PBS (0.087±0.031 g ghrelin *vs*0.056±0.020 g PBS on hypercaloric diet, p=NS; 0.156±0.032 g ghrelin *vs* 0.062±0.016 g PBS on standard diet, p=0.019), in agreement with what has been previously reported (19).

#### 3.2.3 Anti-ghrelin Antibodies Titre Assessment

**Fig. (4):**
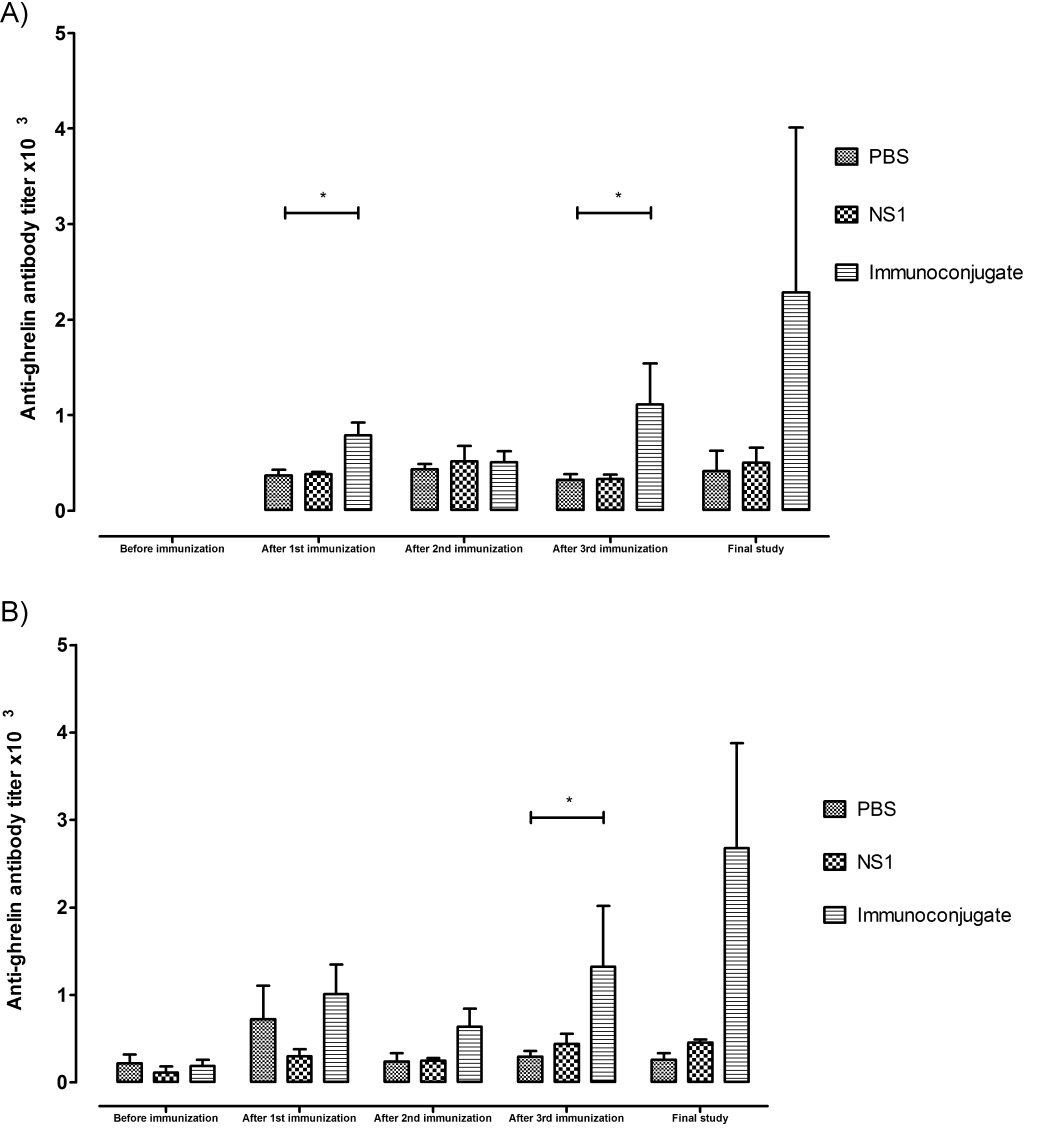
Anti-ghrelin antibody titres of normal weight (*p=0.035) (**A**) and DIO mice (*p=0.03) (**B**) after the immunizations and two weeks after the third immunization. Mice inoculated with the immunoconjugate developed specific anti-ghrelin antibodies in increasing titres, reaching a maximum after the third inoculation in comparison with control groups that maintained constant the basal titres.

Normal weight mice inoculated with the immunoconjugate developed specific anti-ghrelin antibodies, with increasing titres after each inoculation, reaching a maximum of 1265±492 two weeks after the last inoculation. The control groups that received either NS1 protein alone or PBS presented basal titres of 332±114 and 324±143, p=0.035, respectively, which were maintained throughout the study and were not altered by the immunizations, which suggests nonspecific bindings related to the detection method (Fig. **4A**).

DIO mice inoculated with the immunoconjugate also developed specific anti-ghrelin antibodies in increasing titres until reaching a maximum after the third inoculation in contrast with control groups that maintained their basal titres (2680±1197NS1-Ghr, 458±31 NS1 and 257±78 PBS group, respectively, p=0.03) (Fig. **4B**).

#### 3.2.4 Energy Expenditure

Energy expenditure was significantly higher in normal weight mice inoculated with immunoconjugate when compared with controls (0.0146±0.001 kcal/h/kg NS1-Ghr, 0.0138±0.001 kcal/h/kg NS1, 0.0129±0.001 kcal/h/kg PBS, p=0.038).

DIO mice inoculated with the immunoconjugate also showed higher energy expenditure when compared to the control groups (0.0207±0.01 kcal/h/kg NS1-Ghr, 0.0140±0.002 kcal/h/kg NS1, 0.0159±0.002 kcal/h/kg PBS; p=0,044, NS1-Ghr vs PBS and p=0,008, NS1-Ghr vs NS1). 

#### 3.2.5 Hormone Measurements 

Fasting plasma levels of active ghrelin were significantly higher in normal weight mice that received the immunoconjugate (361.3±79.9 pg/ml) when compared to control groups (186.9±14.8 pg/ml NS1 and 114.1±27.9 pg/ml PBS, p=0.009). 

DIO mice inoculated with the immunoconjugate also presented higher levels of fasting plasma ghrelin than the controls (429.63± 179.27 pg/ NS1-Ghr, 147.29±53.17 pg/ml NS1, 105.88±27.76 pg/ml PBS, p=NS) although not statistically significant. There were no significant differences in plasma levels of leptin, insulin, glucose, growth hormone, IGF-1, or TNF-α between the groups (Table **1**). 

#### 3.2.6 Immune Complexes in Circulation and in the Kidney

ELISA confirmed the presence of circulating immune complexes of ghrelin-anti-ghrelin antibodies in the plasma of normal weight mice inoculated with the immune conjugate. There was also a positive correlation between ghrelin plasma levels and the titre of circulating immune complexes (r=0.846) (Fig .**5**). Search for immunoglobulins deposits in the kidney by immunohistochemistry failed to reveal any evidence of deposited immune complexes on the glomerular basement membranes.

#### 3.2.7 Expression of Ghrelin mRNA in the Gastric Fundus and of NPY and POMC in the Basal Hypothalamus

In normal weight mice, there was no significant difference in ghrelin expression in the gastric fundus between the three experimental groups of mice (0.94±0.17 NS1-Ghr, 1.79±0.35 NS1, 1.00±0.30 PBS, p=NS). There was also no significant difference in NPY expression in the basal hypothalamus between the study groups (1.32 ± 0.17 NS1-Ghr, 0.94 ± 0.10 NS1, 1.00 ± 0.20 PBS, p=NS). In contrast, POMC mRNA expression was significantly lower in mice inoculated with the immunoconjugate when compared to controls (0.20±0.17 NS1-Ghr, 0.93±0.17 NS1, 1.00±0.10 PBS, p<0.05).

In DIO mice the expression of ghrelin after normalization for GADPH expression in stomach cells was also not significantly different among the different study groups (1.27±0.30 NS1-Ghr, 0.38±0.13 NS1,1.00±0.12 PBS, p=NS). However, DIO mice inoculated with the immune conjugate had a lower expression of NPY in the basal hypothalamus when compared to control groups (0.59± 0.09 NS1-Ghr, 1.03±0.12 NS1, 1.00±0.13 for PBS, p<0.05). The expression of POMC in the basal hypothalamus was not significantly different between the different groups in study (1.04±0.14 NS1-Ghr, 1.32±0.25 NS1, 1.00±0.12 PBS, p=NS).

## 4 DISCUSSION

Obesity is nowadays a major public health problem [20, 21] for which there is a lack of medical therapeutic resources [6, 22].

Ghrelin is a gastro-intestinal hormone that promotes food intake and decreases energy expenditure [23]. Ghrelin acts in the arcuate nucleus of the basal hypothalamus, stimulating the production and release of NPY and suppressing POMC [24]. NPY is the most potent signal in the central nervous system that stimulates food intake and decreases energy expenditure, while POMC is a precursor protein that through proteolytic cleavage originates various peptides, among which α-MSH that decreases appetite and increases energy expenditure [25-28]. 

Since ghrelin is the only orexigenic hormone identified so far, it has been pointed as a promising treatment target for obesity [29]. Several research groups have previously attempted ghrelin neutralization. Passive transfer of monoclonal anti-ghrelin antibodies was unable to change long-term food intake in mice [11]. Antibodies targeted to hydrolyze the octanoyl moiety of ghrelin to form desacyl ghrelin, which has no biological activity, resulted in increased metabolic rate and suppressed 6h re-feeding after 24h of food deprivation in mice, but this approach would imply the need of periodic antibodies administration [30]. More recently, another study concluded that an oligoclonal response is required to maintain increased energy expenditure during fasting and deprivation-induced food intake as well as to reduce overall food intake upon refeeding [12]. Ghrelin receptor antagonists have also been tested, and GSH-R1a decreased food intake, body weight and improved glucose tolerance due to increased glucose-dependent insulin secretion [13]. Anti-ghrelin vaccines using KLH or BSA as immunogenic substances decreased body weight gain by decreasing feed efficiency in rats [14] and food intake and body weight in pigs [15]. However, these anti-ghrelin vaccination and neutralization strategies present several limitations when applied to humans because of the need to use adjuvants, the risk of exacerbated immune response against an endogenous substance and in the case of passive immunization, acquired tolerance and lack of long-term effect. When compared with classic immunization techniques, VLPs are safe due to the lack of genetic material, since VLPs consist of viral proteins only that prevents a possible reversion to a pathogenic phenotype, induce an efficient B cell activation, since the highly repetitive nature of these structures has the advantage of allowing B cell receptor cross-linking due to the ordered presentation of epitopes in molecule surface, and a high immunogenicity regardless of the route of the immunization, which allows the use of a low number of immunizations and a lower quantity of vaccine, making this type of vaccination protocol more efficient and cost-effective [31]. 

The main goal of the current study was to develop a safer and more effective anti-ghrelin vaccine that could be used for human treatment. For that we developed an immunoconjugate composed of ghrelin and NS1 protein of BTV. The choice of NS1 tubules as VLP-like carrier protein was driven by its previous use as a distribution system for molecules of prophylactic vaccines against common human infectious diseases, such as proteins of the foot-and-mouth disease and influenza A virus [19, 32].

The ability of the vaccine to trigger an immune response was tested in normal weight and DIO male mice that developed increasing titres of specific anti-ghrelin antibodies, confirming the hypothesis that a vaccine consisting of immunoconjugate only is able to trigger an immune response without the need adjuvants. Furthermore, antibodies titres attained after the immunization protocol were not very high, when compared to antibodies titres after common infectious diseases, which is also reassuring in safety concerns, since complete neutralization of ghrelin was not the purpose of an anti-ghrelin vaccination strategy for obesity treatment as ghrelin also intervenes in several key biological processes besides appetite regulation, such as growth hormone secretion, gastro-intestinal and cardiovascular functions [29]. In the present study we chose to use only male mice, to allow the characterization of the effects of the vaccine irrespective of other hormonal influences such as the ovarian cycles. The decision to use diet induced obesity mice (DIO) that develop obesity in result of the sedentary lifestyle and increased caloric intake, resided in the fact that this obesity animal model is the one that more accurately reflects the most common cause of obesity in order to allow the evaluation of the interaction between ghrelin and anorexigenic pathways, which compared to the ob/ob or the db/db mouse, which present monogenetic causes of obesity that are rare in the human setting. 

Vaccinated mice presented acute decreases in food intake in the 24h immediately after the immunizations that were more pronounced in DIO mice. This acute decrease in food intake, cannot be attributed to an immune response given the short time elapsed after the inoculation; and because mice presented no signs of sickness or changes in TNF-α levels, an alternative explanation could be a sequestration of free plasma ghrelin by the immunoconjugate. Although unlikely, as the excess of crosslinker was chemically neutralized before inoculating the mice. Since mice daily and cumulative food intake over the study time span was not significantly different in vaccinated mice compared to controls, it suggests the need for optimizing the immunization protocol herein used, namely the time gap between the inoculations and the amount of anti-ghrelin vaccine administration.

Vaccinated mice showed significantly higher energy expenditure than the animals of the groups that received either NS1 protein alone or PBS. Higher energy expenditure usually translates into greater ease of weight loss and maintenance. Ghrelin is known to suppress energy metabolism and ghrelin replacement partially reverses the reduction in body weight and body fat in gastrectomised mice [33]. Ghrelin has been shown to have a long-term effect on energy homeostasis by increasing the respiratory quotient, through decreasing utilisation of fat as energy [34]. In addition, ghrelin knockout mice compared to wild type mice present no change in food intake but have a decreased respiratory quotient when fed with high fat diet, suggesting that endogenous ghrelin plays a more prominent role in determining the type of metabolic substrate that is used for maintenance of energy balance, than in the regulation of food intake [35]. Although vaccinated animals gained less weight when compared with control animals, this difference failed to reach statistical significance, which may be explained by the short follow-up time or the activation of compensatory mechanisms of energy homeostasis pathways. 

Paradoxically, vaccinated mice had higher ghrelin levels compared to controls. Given that these increased levels of ghrelin did not appear to have a biological effect, we hypothesize that circulating ghrelin could be in the form of immune complexes of ghrelin-anti-ghrelin, which was confirmed. Previous reports on anti-ghrelin vaccines have also documented an increase of ghrelin in immunized animals, although the presence of circulating immunocomplexes has not been documented [36]. The presence of circulating immunocomplexes, which could be due to a lower rate of elimination, raised the concern of renal toxicity due to the deposition in the glomerular basement membrane that has been excluded. Since there was no difference in ghrelin expression in the stomach, ghrelin appears to be synthesized in immunized animals as in controls and after neutralization of ghrelin biological activity there is no up regulation of ghrelin expression in order to maintain the homeostasis.

In vaccinated normal weight mice there were no significant differences in the genetic expression of NPY gene in the basal hypothalamus in comparison to control mice. However, in vaccinated DIO mice there was a significant decrease of NPY gene expression in the basal hypothalamus compared with controls reflecting a decrease in central orexigenic signals [37]. The expression of POMC in the basal hypothalamus was significantly lower in vaccinated normal weight animals compared to controls that could represent a compensatory mechanism to the decreased peripheral orexigenic signals in order to prevent the reduction in feeding threshold of normal weight mice, which could also explain why these findings only occurred in the normal weight mice but not on DIO mice.

Ghrelin is a growth hormone secretagogue [29] and ghrelin neutralization could induce alterations in GH/IGF-1 axis. Since this vaccine appears to have no effect in GH and IGF-1 levels, this suggests that our vaccine is unlikely to cause endocrine adverse effects on the growth hormone axis.

The regulatory mechanisms of energy homeostasis and appetite control are very complex processes that include highly redundant signalling pathways [24].Therefore, it is possible that the lack of significant differences in some biological parameters, such as food intake and body weight maybe due to activation of compensatory mechanisms for the decrease in available active ghrelin similar to that which occurs in ghrelin knockout mice [35, 38].

One of the major acquisitions of the research work described in this paper was the demonstration of the capacity of this vaccine to efficiently induce the production of antibodies against an endogenous molecule without the requirement to use adjuvants and the ability to neutralize some of ghrelin biological effects. Furthermore, this vaccine appears to be well tolerated by the animals and there were no signs of inflammatory reaction or toxicity. The production of anti-ghrelin antibodies was effective in decreasing acute food intake and increasing energy expenditure in the vaccinated animals compared to control animals, which are important contributions to establish a negative energy balance and thus promote weight loss.

## CONCLUSION

In conclusion, these results suggest that this anti-ghrelin vaccine has a positive impact on energy homeostasis and may be a useful tool for obesity treatment. 

## Figures and Tables

**Fig. (5):**
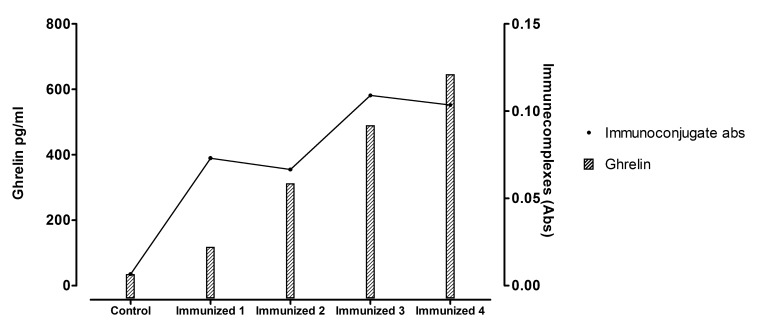
Circulating immune complexes titres and plasma ghrelin levels. There was a positive correlation between ghrelin plasma levels and the titre of circulating immune complexes (r=0.846).

**Table 1.** Fasting hormone and glucose plasma levels of normal weight and DIO mice.

	Normal weight mice				DIO mice			
	PBS	NS1	Vaccine	Sig.	PBS	NS1	Vaccine	Sig.
Ghrelin (pg/ml)	114.1 ± 27.9	186.9 ± 14.8	361.3 ± 79.9	p=0.009	105.9 ± 27.8	147.3 ± 53.2	429.6 ± 179.3	NS
Leptin (ng/ml)	1.32 ± 0.23	0.98 ± 0.21	1.50 ± 0.61	NS	5.85 ± 0.93	5.15 ± 0.75	7.11 ± 0.39	NS
Insulin (ng/ml)	0.35 ± 0.08	1.08 ± 0.63	0.41 ± 0.07	NS	1.31 ± 0.09	1.01 ± 0.13	1.27 ± 0.11	NS
Growth hormone (ng/ml)	2.65 ± 0.69	5.50 ± 2.44	2.57 ± 0.41	NS	2.36 ± 0.74	5.28 ± 1.95	4.13 ± 1.31	NS
IGF-1 (ng/ml)	473.8 ± 68.6	561.7 ± 35.3	499.8 ± 62.1	NS	347.7 ± 17.9	387.4 ± 34.5	394.8 ± 21.1	NS
Glucose (mg/dl)	86.5 ± 6.3	82.3 ± 8.2	76.3 ± 2.9	NS	156.3 ± 31.8	128.2 ± 15.0	121.8 ± 11.3	NS
TNF-α	0.30 ± 0.30	8.42 ± 4.22	1.65 ± 1.31	NS	45.59 ± 24.72	5.76 ± 4.44	21.34 ± 15.29	NS
